# The Content Validity of the Cultural Formulation Interview (CFI)

**DOI:** 10.1155/2018/3082823

**Published:** 2018-12-02

**Authors:** Behnam Shariati, Amir-Abbas Keshavarz-Akhlaghi, Arash Mohammadzadeh, Ruohollah Seddigh

**Affiliations:** ^1^Mental Health Research Center, Rasoul-e-Akram Hospital, Iran University of Medical Sciences, Tehran, Iran; ^2^Mental Health Research Center, Tehran Institute of Psychiatry, School of Behavioral Sciences and Mental Health, Iran University of Medical Sciences, Tehran, Iran; ^3^Spiritual Health Research Center, Iran University of Medical Sciences, Tehran, Iran

## Abstract

**Aims:**

The present study investigates content validity of the open-ended items in Cultural Formulation Interview in the Diagnostic and Statistical Manual of Mental Disorders, 5th Edition (DSM-5).

**Methods:**

After translating into Persian and assessing the content validity of the items and their modification by psychiatrists, the questionnaire was translated back into English by two translators and then retranslated into Persian. The final Persian version and its back-translation were submitted for approval to the DSM-5 design workshop in the United States. After obtaining the group's approval on the back-translation and permission to use the questionnaire, samples were distributed among panel members and the content validity of the questionnaire was thus examined.

**Results:**

The content validity index (CVI) of cultural formulation interview was 0.51 and all the items were acceptable although some, especially those on the cultural perception of the context and the cultural factors affecting current help-seeking, had lower content validity ratios (CVR).

**Conclusion:**

The cultural formulation interview seems to have an overall acceptable content validity although it is weak and thus needs further studies in relation to the two domains of the cultural perception of the context and the cultural factors affecting help-seeking in the Iranian population.

## 1. Introduction

The cultural formulation of psychiatric disorders is important in many different ways, including the prevention of misdiagnoses as discussed by Adeponle and elsewhere [1, 2], obtaining crucial clinical information [3–5], improving the patient rapport and interaction [6], improving therapeutic effectiveness [7], and serving as a guide to clinical studies [8] and cultural epidemiological studies [9] on psychiatric disorders. After introducing the outline for cultural formulation, a lot of studies were conducted on the concept and an open-ended questionnaire was proposed in the DSM-5 on cultural evaluation within different dimensions, namely, the Cultural Formulation Inventory (CFI) [10]. This inventory investigates four cultural components pertaining to psychiatric disorders, including the cultural definition of the problem, the cultural perception of the cause, the cultural factors affecting self-coping, and the cultural factors affecting current help-seeking [10].

Given the inadequate attention to this increasingly important subject in psychiatric education and treatment in Iran and its introduction into the official resources and classifications of psychiatry, conducting studies to investigate it appears beneficial. Furthermore, the cultural differences within the country add to the importance of assessing the content validity of the inventory prior to its use in clinical settings.

## 2. Materials and Method

The CFI was introduced into the classification book of the DSM-5 in 2013 [10] in two forms, including the main form, which is filled out by the patient and was the form that was investigated in the present study, and the other form for informants, which is quite similar to the first form, except with minor spelling modifications, and is filled out by the patients' caregivers. The CFI assesses four cultural components pertaining to psychiatric disorders, including the cultural definition of the problem (items 1-3), the cultural perception of the cause, context and support (items 4-10), the cultural factors affecting self-coping and past help-seeking (items 11-13), and the cultural factors affecting current help-seeking (items 14-16).

The questionnaire was first translated into Persian by three assistant professors of psychiatry and the translated questionnaire was then submitted to a panel of 20 experts for examining its face validity. The proposed changes were then implemented by three faculty members and were approved by the panel members. It was then translated back into English by two translators with a PhD in English translation and the final Persian version as well as its back-translation was then submitted to the DSM-5 design workshop in the US. After obtaining the group's approval for the back-translation and permission to use the questionnaire, it was distributed among the panel members in order to have its content validity assessed.

### 2.1. Content Validity Assessment

Holsti describes content validity assessment as a technique for making inferences through the objective and systematic identification of specific characteristics of a message [11] In order to determine the content validity of the questionnaire, the method proposed by Chadwick et al. and Lawshe [12, 13] was used. Chadwick et al. argue that the content validity method can be used when a means of information exchange containing relatively clear and inferential messages is meant to be introduced and explained in practice. Lawshe recommends that researchers use the content validity method when high levels of abstraction and insight are needed for a judgment and when a wide range of inferences can be made about the content of a message.

In Lawshe's method of content analysis, a panel of experts first comments on whether the components of the tool are essential, and their comments are recorded as essential (E), useful but not essential (U), and not necessary (N) [12].

### 2.2. Modifying the Selected Pattern

As the scale in question can be interpreted differently based on Lawshe's encoding method, even while taking into account the instructions given by Henderson and M, Freeman CP [14], the researchers decided to rate the questionnaire items based on a scale consisting of ‘completely agree', ‘agree', ‘no comments', ‘disagree', and ‘completely disagree'.

As per Leedy and Ormrod's instructions, using this scale appears to facilitate the responding procedure by proposing a wider range of responses and adding the option of ‘no comments' [15]. Moreover, being a Likert-type scale, the sequential arrangement of the responses is more evident with the use of this scale. The instructions at the beginning of the questionnaire also asked the members to record their proposed modifications for the items with which they “disagreed” or “completely agreed”; they were thus able to add more dimensions and items to the questionnaire.

### 2.3. Choosing Panel Members for the Validity Assessment Task

The panel members were chosen in this stage from among those active in the content range of measures and based on predetermined goals and so as to enable a proper assessment of the questionnaire. A limited number of members were first introduced as the heads of the groups and they then helped introduce the other members of the panel. Although the minimum number of group heads is four based on Lawshe's method, 32 members were chosen so as to overcome problems such as participant withdrawal and unreturned questionnaires and to also enhance the reliability of the results.

### 2.4. Questionnaire Distribution and Collection

The panel members were contacted in person, by phone, or via email. Of the total of 32 questionnaires, 20 were ultimately filled out, making for a return rate of 62.5%.  Table 1 presents the members' details.

### 2.5. Data Entry

Microsoft Excel was used to analyze the mathematical and statistical calculations made by the panel members for the assessment.

### 2.6. Quantification of the Panel Members' Comments through Calculation

The following formula was used to calculate the CVR for the panel members' ‘E' votes [12].

(1) $$\begin{array}{c} CVR=\frac{ne-N/2}{N/2} \end{array}$$

ne is the number of the panel members who rated this dimension or item as essential (‘E”='), and N/2 is the total number of group members divided by two.

The values assigned to CVR are as follows:

(1) Negative when less than half the members choose ‘E'

(2) One when all the members choose ‘E'

(3) Between 0 and 99% when more than half but less than 100% of the members choose ‘E'

The following assumptions can be interpreted based on the CVRs [12]:

(1) When all the panel members rate an item as not essential (‘U'), the item is interpreted as completely unessential.

(2) When all the members rate an item as U, two scenarios can be true: either all are wrong or all are right. As the panel is made up of experts, the assumption is that they are not all wrong, so the item is deemed significantly essential.

(3) For all the cases in between, the item rated as U by more than half of the members has a certain degree of content validity and the higher is the number of panel members rating the item as U, the higher is the content validity of the item.

### 2.7. The Mean Values Assigned by the Panel Members

According to Lawshe's method [12], the following conversions are made in the validity assessment questionnaire to calculate the mean values assigned to each component of the tool:

  ‘E', which shows that an item is essential, is replaced with 2.
  ‘N', which shows that an item is useful but not essential, is replaced with 1.
  ‘U', which shows that an item is not essential, is replaced with 0.

Only the components with CVRs and mean values that are consistent with the acceptable minimum values remain in the final questionnaire.

As was noted before, since the present study used a 5-point Likert-type scale (‘completely agree', ‘agree', ‘no comments', ‘disagree', and ‘completely disagree') instead of the 3-point Lawshe scale to obtain better results, the proper adaptation of the scale required the nominal rating criteria to be converted to Lawshe's numerical rating criteria as follows:

  ‘Completely agree' and ‘agree' (taken to refer to ‘essential') were replaced with 2.
  ‘No comments' (taken to refer to ‘useful but not essential') was replaced with 1.
  ‘Disagree' and ‘completely disagree' (taken to refer to ‘not essential') were replaced with 0.

## 3. Item Acceptance or Rejection Criteria

(1) The item is accepted unconditionally if CVR is equal to or higher than 0.42, calculated based on the number of panel members.  Table 2 presents the details.

(2) The item is accepted if its CVR is between 0 and 0.42, i.e., more than half of the members have chosen the ‘completely agree' or ‘agree' option, and the mean numerical rating is equal to or higher than 1.5, i.e., the mean rating is closer to the ‘completely agree' and ‘agree' options and the mean rating is equal to or higher than 75% of mean of 2, which is higher than the minimum 60% set for acceptable content validities [13].

(3) The item is rejected if CVR is less than 0 and the numerical mean of the items is less than 1.5, i.e., less than half of the members have chosen the ‘completely agree' or ‘agree' options and the mean numerical rating is closer to the ‘no comments' option.

## 4. Determining the CVI

CVI is calculated according to the following equation and is taken to indicate the comprehensiveness of the assessment of the validity or applicability of the model, test, or final tool.

(2) $$\begin{array}{c} CVI=\frac{\sum_{1}^{n}CVR}{Retained\;\;numbers} \end{array}$$

CVR is the linear and direct form of conversion of the panel members who have chosen the ‘U' option.

‘Retained numbers' is the number of items remaining.

## 5. Results

Items 2, 8, 9, 10, 12, 15, and 16 were acceptable while all the other items were unconditionally acceptable. The CVR was 0.2 for items 8, 9, and 10, 0.3 for items 12, 15, and 16, 0.4 for item 2, and 0.5 or higher for all the other items. The CVI was 0.51 for the entire scale.  Table 3 presents the details.

## 6. Discussion

Based on the CVRs obtained in the present study, all the items can be included in the final version of the Iranian Cultural Formulation Interview; however, the results obtained require more clarification.

As can be witnessed, the items pertaining to the four cultural components do not have the same content validity. Items 8, 9, and 10 on the cultural perception of the context and items 15 and 16 on the cultural factors affecting current help-seeking have a lower content validity for adaptation to the Iranian culture and should be further addressed. Moreover, the scores obtained for these items have lowered the overall CVI of the scale to around 0.5, which differs significantly from the 0.7 score set by Chadwikk as an acceptable validity for questionnaires [13]. Omitting these five items increases the scale's CVI to 0.63, which is a relatively good score.

The low scores of these two domains can be justified by noting that the inability of items 8, 9, and 10 to identify the cultural context may be due to the so-called collective culture of Iran. The Iranian culture is taken to resemble the South Asian cultural cluster of India, Malaysia, and Thailand, which possess both strong family ties and a great level of individualism. These cultures attribute a high value and status to those in power, do not tolerate dissidence, do not welcome collective support and opinions, and have remained intact despite the great social changes occurred in recent decades [16]. Numerous studies have demonstrated the great effectiveness of culture in identifying the cultural factors at play in the development of disorders [17]. Moreover, many factors affect the decision to seek help and define the problem, including gender, social status, and cultural factors such as the attitude toward men and women and their roles as discussed by Liang and elsewhere [18, 19]. Defining the cultural components affecting the definition of disorder and understanding the contributing cultural factors affect disorder and turn it into a very complicated issue, which may justify the inadequacy of items 8, 9, and 10 in distinguishing these issues. The low scores obtained for items 15 and 16 may be justified by examining cultural factors, particularly gender and type of disorder according the Vahdaninia's study [20], the type of authoritarian culture in place, which prohibits external help [16], and even the particular disorder formulation among Iranian people, who do not welcome external help and believe mental disorders to be caused by external factors and in response to social issues and, to a lesser extent, to family problems; for example, Kurd and Turk people ask for professional help less than Fars people and rely more on therapists and traditional support for help. In addition, social stigma and its severity are also a crucial cultural factor at play [21, 22]. The beliefs of every culture and even subculture, such as Turk people against Fars people, affect the concept of virility and fertility in the formation of psychiatric disorders and the type of help-seeking for the purpose of treatment [23]. Moreover, Iranian women attribute mental health to numerous factors; for example, they regard marital satisfaction as a significant factor and identify religion as a key factor in mental health [24]. Research suggests that, in different Iranian ethnicities, especially in Kurd people, geographical location and people's perception of social threats such as war affect their conceptualization of psychiatric disorders [25].

Overall, further studies are recommended to be conducted on this questionnaire, as it seems to be less adapted to the Iranian population in the two domains of the cultural perception of the context and the cultural factors affecting help-seeking.

## 7. Conclusion

The cultural formulation interview seems to have an overall acceptable content validity although it is weak and thus needs further studies in relation to the two domains of the cultural perception of the context and the cultural factors affecting help-seeking in the Iranian population.

## Figures and Tables

**Table 1:** Details of the 20 members of the panel of content validity assessment.

**Details**	**Academic Rank**	**Number**
PhD in Counseling Psychology	Associate professor	1
PhD in Clinical Psychology	Associate professor	2
PhD in Theology and Islamic Sciences	Two assistant professors and one nonfaculty member	3
Psychiatrist	One professor and one assistant professor	2
PhD in Sociology	Associate professor	1
PhD in Social Work	Associate professor	2
MSc in Psychiatric Nursing	Medical staff	3
Patient		4
Social Medicine Specialist	Assistant professor	2

Total	20	

**Table 2:** The acceptable CVRs corresponding to the number of panel members.

Number of Panel Members	Minimum Acceptable CVR
5	99%
6	99%
7	99%
8	78%
9	75%
10	62%
11	59%
12	56%
13	54%
14	51%
15	49%
20	42%
25	37%
30	33%
35	31%
40	29%

**Table 3:** CVI and CVR of the Persian version of the Cultural Formulation Interview.

**Questions of CFI(briefly)**	**CVR**	**Mean numerical rating**	**Acceptable or rejection**	**CVI**
(1) What brings you here today?	**0/9**	**1/9**	unconditionally acceptable	

(2) People sometimes describe their problem in different ways for family, friends, or other persons. How would you describe your problem for them?	**0/4**	**1/7**	acceptable	

(3) Which aspect of your problem troubles you more?	**0/5**	**1/75**	unconditionally acceptable	

(4) Why did this problem happen to you? What are the causes?	**0/5**	**1/75**	unconditionally acceptable	

(5) What do significant people in your life (e.g., family members, friends, or other acquaintances) think about the reason of your problem? (or what do they say?)	**0/9**	**1/9**	unconditionally acceptable	

(6) Is there any kind of support that can alleviate your problem (Can others help you to resolve your problem), such as the support of family, friends, or others?	**0/9**	**1/9**	unconditionally acceptable	

(7) Is there any psychological stress that worsens your problem, such as financial problems or family problems?	**0/8**	**1/8**	unconditionally acceptable	

(8) What are the most important aspects of your cultural background or identity?	**0/2**	**1/6**	acceptable	

(9) Is there anything with a major impact on your problem in your cultural background or identity?	**0/2**	**1/5**	acceptable	

(10) Is there anything creating concerns or other problems for you about your cultural background or identity?	**0/2**	**1/6**	acceptable	

(11) Sometimes, people use different ways to cope with their problems. What methods have you used personally to cope with your problems?	**0/6**	**1/8**	unconditionally acceptable	

(12) People often get help from many different individuals, e.g., different physicians or healers. What types of treatment, help, advice, or healing have you used to solve your problem in the past?	**0/3**	**1/65**	acceptable	

(13) Has anything prevented you from receiving help?	**0/6**	**1/75**	unconditionally acceptable	

(14) What kinds of help will be most useful for you at the current situation and in relation to your problem?	**0/6**	**1/8**	unconditionally acceptable	

(15) Are there other help resources useful for you according to your family, friends, or other acquaintances at the current situation?	**0/3**	**1/6**	acceptable	

(16) Do you have concerns about this issue, and is there anything we can do to provide better services for you?	**0/3**	**1/55**	acceptable	

CVI				**0/51**

## Data Availability

The data used to support the findings of this study are available from the corresponding author upon request.
